# Rules in Case of Fire

**Published:** 1883-08

**Authors:** 


### RULES IN CASE OF FIRE.

Our actions in sudden danger are apt to be mostly illustrations of "how not to do it." It is only calm good sense that gets away whole and sound in such emergencies, while fright turns somersaults and steps on his own head. The experience of others has given us rules of the best procedure when we find ourselves in a "house-afire;" and a knowledge of these beforehand will be of great use to us, if we can only keep our wits about us when the time comes.

In case of either a chimney or a room catching fire, the first thing to be thought of is to exclude all draughts, for it is certain that the slightest current of air will increase the force of the fire.

All the doors and windows should be shut at once, and if the chimney should be on fire, a wet blanket should be immediately fastened to the top of the mantel-piece, so as to exclude all draughts from the opening of the chimney, and entirely cover the grate, shutting the trap first if possible.

This will, in most cases, make the fire go out of itself. You can throw into the grate a few handfuls of salt. Water should never be thrown down from above, as it spoils the carpet and furniture unnecessarily.

If the window or bed-curtains catch fire, beat them with the thickest woolen garment you can lay your hands upon. Window-curtains can in most cases be torn down with a violent jerk; and this will prevent the flames from extending to the woodwork of the windows. In escaping from a burning house or room, remember that the air nearest the floor is clearer than any, and go on your hands and knees at once.

A wet cloth tied over the mouth and nose keeps out the smoke, will help the breathing, and prevent suffocation if too much oppressed.

A wet blanket, or even a dry one speedily used, will extinguish many a small conflagration—such, for instance, as of an upset lamp, by excluding the air, and will be far more efficacious than water thrown for that purpose; its use also prevents damage to furniture.

When an alarm of fire is given, if in bed, wrap yourself in a blanket, which will form the best protection for you from the chance of ignition, and endeavor to remember the different exits from the house—where they are and how to reach them; if you cannot attain to any of them, try to get to a front room as near the ground as possible.—*Leisure Hours.*

### FOOD ADULTERATION.

The evils resulting from the use of Adulterated Baking Powders culminated a few years ago in a popular war against the products of manufacturers who substituted Alum for Cream of Tartar.

Whenever public feeling becomes aroused against an evil there is usually a strong effort made to abate it; and so, in this instance, almost the entire community seemed to protest against being longer fed on a questionable article that found its way into "the staff of life;" but it seems impossible to wholly destroy a business conducted by heartless men, and that affords enormous profits. The only way is to warn people against the use of adulterated and poisonous preparations of every sort, and make our warning effective by stating what preparations are pure and reliable. This is a matter within the province of the JOURNAL OF HEALTH, and an honest statement of the facts, without fear or favor, is due to its readers. We say then: as you value your health, and the health of those under your care, avoid the use of all foods, candies, baking powders, and other articles, whose purity is questionable.

There is also a choice in the products put into the market by reputable manufacturers.

The best baking powder is made from pure Cream of Tartar, Bicarbonate of Soda, and a small quantity of flour or starch. Frequently other ingredients are used, and serve a purpose in reducing the cost and increasing the profits of the manufacturer.

We give the Government Chemist's analyses of two of the leading baking powders:

I have examined samples of "Cleveland's Superior Baking Powder" and "Royal Baking Powder," purchased by myself in this city, and I find they contain:

**"Cleveland's Superior Baking Powder."**

Cream of Tartar.
Bicarbonate of Soda.
Flour.

Available carbonic acid gas 12.61 per cent., equivalent to 118.2 cubic inches of gas per oz. of Powder.

---

**"Royal Baking Powder."**

Cream of Tartar.
Bicarbonate of Soda.
Carbonate of Ammonia.
Tartaric Acid.
Starch.

Available carbonic acid gas 12.40 per cent., equivalent to 116.2 cubic inches of gas per oz. of Powder.

Ammonia gas 0.43 per cent., equivalent to 10.4 cubic inches per oz. of Powder.

Note.—The Tartaric Acid was doubtless introduced as free acid, but subsequently combined with ammonia, and exists in the Powder as a Tartrate of Ammonia. E. G. LOVE, Ph.D

NEW YORK. Jan. 17th, 1881.

The above analyses indicate a preference for "Cleveland's Superior Baking Powder," and our opinion is that it is the better preparation.

Problem of the Universe.—"If asked," says Prof. Tyndall, "whether science has solved, or is in our day likely to solve the problem of the universe, I must shake my head in doubt. Behind and above and around us the real mystery of the universe lies unsolved, and, as far as we are concerned, is incapable of solution The problem of the connection of the body and the soul is as insoluble in its modern form as it was in the prescientific ages. There ought to be a clear distinction made between science in the state of hypothesis and science in the state of fact ; and inasmuch as it is still in its hypothetical stage, the ban of exclusion ought to fall upon the theory of evolution."

Accidents to the Teeth.—Boys, and even men, occasionally lose teeth from blows, or a bungling dentist may extract a sound tooth by mistake. Now, it should be known by everybody, that teeth thus taken out may be replaced exactly in their natural position by any one within an hour or two, or by a skillful dentist after the lapse of twenty-four hours or more. Where much time has elapsed, the tooth must be carefully washed in tepid water, and then covered with glycerine. The cavity is syringed with warm water, containing a few drops of carbolic acid. The main thing is to have the cavity and the tooth thoroughly cleansed, for inclcsing any foreign substance in the cavity with the roots of the tooth might cause ulceration. The tooth is tapped with a hammer to get it into its place. We are acquainted with a young man who left two front teeth over night on a base-ball ground, and who on learning next day that a dentist could restore them, searched and found them, and they now occupy their places, seemingly as good as ever.

Crooked teeth may be taken out and put back so as to be even.

Bleeding to Death.—The large arteries are at considerable depth from the surface of the body, so as to be protected from accident; still the flesh offers but little resistance to cutting instruments, and wounds to the arteries are of frequent occurrence, particularly among children. The blood in the arteries flows from the heart and when an artery is opened the red blood spurts out at each pulsation. All that is necessary to do in case of such an accident is to produce enough pressure on the artery, at any point between the wound and the heart, to stop the flow. This may generally be done by pressing the end of the thumb against the artery above the wound, or into the wound itself ; or, tie a handkerchief around the limb, and make it tight by twisting it with a stick. Do just what you would do to prevent water from flowing out of a small rubber tube. No person with a cool head, who remembers these simple instructions, would ever let a patient die of such a wound in his presence ; yet thousands of lives have been sacrificed for want of this knowledge.

SOME USES OF CHARCOAL.—Charcoal, laid flat while cold on a burn, causes the pain to abate immediately ; by leaving it on for an hour the wound seems almost healed, when the wound is superficial. Tainted meat surrounded with it is sweetened. Foul water is purified by it. It is a great disinfectant, and sweetens offensive air if placed in shallow pans around apartments. It is so very porous that it absorbs and condenses gases rapidly. One cubic inch of fresh charcoal will absorb nearly one hundred inches of gaseous ammonia. Charcoal forms an excellent poultice for malignant wounds and sores. In cases of what is called proud flesh it is invaluable. It gives no disagreeable odor, corrodes no metal, hurts no texture, injures no color, is a simple and safe sweetener and disinfectant A teaspoonful of charcoal in half a glass of water often relieves a sick headache. It also relieves constipation, pain or heartburn.

"Do NOT understand me at all," said Prof. C. A. Young in a late astronomical lecture, "as saying there is no mystery about the planets' motion. There is just one single mystery—gravitation—and it is a very profound one. How it is that an atom of matter can attract another atom, no matter how great the disturbance, no matter what intervening substance there may be; how it will act upon it, or at least behave as if it acted upon it, I do not know, I cannot tell. Whether they are pushed together by means of an intervening ether, or what is the action, I cannot understand. It stands with me along with the fact that when I will my arm to rise, it rises. It is inscrutable. All the explanations that have been given of it seem to me merely to darken counsel with words and no understanding. They do not remove the difficulty at all. If I were to say what I really believe, it would be that the motion of the spheres of the material universe stand in some such relation to Him in whom all things exist, the ever present and omnipotent God, as the motions of my body do to my will—I do not know how, and never expect to know."

FRIGHTENED TO DEATH.—The London *Daily News* mentions a "joke" which had a fatal termnation, and comments upon it as follows : "A girl of eighteen, named Harriet Etherington, has just been frightened to death at Brockley. She waswalking on a lonely road beside a cemetery, when a man with somthing white around his face 'flew out at her.' Probably the neighborhood of the graves may have disposed her to be readily alarmed. She went home, told her story and fell down dead at her father's table.

"There is a class of idiots who think it amusing to play on the nerves of women in this manner. To be frightened terribly by a person in a hideous disguise, who leaps out suddenly in the dark, a girl need not be superstitious, or inclined to believe in churchyard spectres. The suddenness of the attack might startle even a man of strong nerve for a moment. To a girl, still more to a child, such an attack may mean simply murder."

